# Synergistic Association of Valproate and Resveratrol Reduces Brain Injury in Ischemic Stroke

**DOI:** 10.3390/ijms19010172

**Published:** 2018-01-06

**Authors:** Lara Faggi, Giuseppe Pignataro, Edoardo Parrella, Vanessa Porrini, Antonio Vinciguerra, Pasquale Cepparulo, Ornella Cuomo, Annamaria Lanzillotta, Mariana Mota, Marina Benarese, Paolo Tonin, Lucio Annunziato, PierFranco Spano, Marina Pizzi

**Affiliations:** 1 Department of Molecular and Translational Medicine, University of Brescia, 25123 Brescia, Italy; l.faggi@unibs.it (L.F.); v.porrini@unibs.it (V.P.); annamaria.lanzillotta@unibs.it (A.L.); m.coelhodamota@unibs.it (M.M.); marina.benarese@unibs.it (M.B.); pierfranco.spano@unibs.it (P.S.); marina.pizzi@unibs.it (M.P.); 2 Department of Neuroscience, Reproductive Sciences and Dentistry, University of Naples Federico II, 80131 Naples, Italy; giuseppe.pignataro@unina.it (G.P.); antonio.vinciguerra@unina.it (A.V.); lino.cepparulo@gmail.com (P.C.); orcuomo@yahoo.it (O.C.); lucio.annunziato@unina.it (L.A.); 3 IRCCS, S. Camillo Hospital, 30126 Venice, Italy; paolo.tonin@ospedalesancamillo.net; 4 IRCCS, SDN, Via Gianturco, 113, 80142 Naples, Italy

**Keywords:** valproate, resveratrol, oxygen glucose deprivation (OGD), middle cerebral artery occlusion (MCAO), RelA

## Abstract

Histone deacetylation, together with altered acetylation of NF-κB/RelA, encompassing the K310 residue acetylation, occur during brain ischemia. By restoring the normal acetylation condition, we previously reported that sub-threshold doses of resveratrol and entinostat (MS-275), respectively, an activator of the AMP-activated kinase (AMPK)-sirtuin 1 pathway and an inhibitor of class I histone deacetylases (HDACs), synergistically elicited neuroprotection in a mouse model of ischemic stroke. To improve the translational power of this approach, we investigated the efficacy of MS-275 replacement with valproate, the antiepileptic drug also reported to be a class I HDAC blocker. In cortical neurons previously exposed to oxygen glucose deprivation (OGD), valproate elicited neuroprotection at 100 nmol/mL concentration when used alone and at 1 nmol/mL concentration when associated with resveratrol (3 nmol/mL). Resveratrol and valproate restored the acetylation of histone H3 (K9/18), and they reduced the RelA(K310) acetylation and the Bim level in neurons exposed to OGD. Chromatin immunoprecipitation analysis showed that the synergistic drug association impaired the RelA binding to the *Bim* promoter, as well as the promoter-specific H3 (K9/18) acetylation. In mice subjected to 60 min of middle cerebral artery occlusion (MCAO), the association of resveratrol 680 µg/kg and valproate 200 µg/kg significantly reduced the infarct volume as well as the neurological deficits. The present study suggests that valproate and resveratrol may represent a promising ready-to-use strategy to treat post-ischemic brain damage.

## 1. Introduction

Stroke is one of the leading causes of mortality and morbidity worldwide with an increasing number of reports and clinical trials performed every year. The only approved pharmacological treatment for ischemic stroke is tissue plasminogen activator (tPA) within 4.5 h from the ischemic event [1]. However, due to the increased risk of hemorrhagic conversion in the infarcted brain, tPA administration after 4.5 h of the stroke onset is contraindicated and generally results in negative outcomes. To date, the identification of a neuroprotective therapy remains an unmet need [2]. Stroke pathogenesis is complex and involves numerous processes including excitotoxicity, oxidative damage, apoptosis and inflammation [3,4,5,6].

In the last few years different studies demonstrated that epigenetic changes occur during brain ischemia. In particular, histone deacetylation and the modification of NF-κB/RelA acetylation seem to be involved in stroke pathophysiology [7,8,9]. Among post-translational modifications of histones, acetylation plays an essential role in the gene activation regulating the accessibility of chromatin to the transcriptional machinery [10]. Acting in an opposite way, histone acetyltransferase (HAT) and histone deacetylase (HDAC) reversibly regulate the acetylation of lysine residues. A general decrease of HAT activity lead to the over-deacetylation described in brain ischemia [11,12], so that application of HDAC inhibitors showed a therapeutic efficacy in mice models of middle cerebral artery occlusion (MCAO) [13,14,15,16,17]. Recently, it has been demonstrated that suppressing HDAC2 in the peri-infarct cortex of rodents promotes the motor function recovery at day 8 after stroke, opening a new therapeutic window for the treatment of stroke [18].

Beside histones, other non-histone proteins that comprise transcription factors such as NF-κB, are targets of HATs and HDACs [19]. NF-κB acts in the central nervous system as a pleiotropic regulator of genes controlling either cell survival [20] or the apoptosis and inflammation associated with neurodegeneration [21,22]. In previous studies we showed that the acetylation state of NF-κB/RelA (RelA) and histones can discriminate protective and neurotoxic effects elicited by NF-κB in brain ischemia [8,9,23]. Either the neuroprotective ischemic preconditioning or the noxious ischemia activated the p50/RelA dimer, but only the ischemic injury induced an atypical RelA acetylation on Lys 310 residue with a statistically relevant reduction of H3 histone acetylation. In parallel with the RelA Lys310 acetylation, the interaction of RelA with the histone acetyltransferase cAMP-response element binding protein CREB-binding protein (CBP)/p300 complex also increased [8]. In primary cortical neurons exposed to oxygen glucose deprivation (OGD) and in the cortex of mice exposed to “MCAO”, these changes were associated with RelA detachment from the anti-apoptotic *Bcl-x_L_* promoter and binding to the pro-apoptotic *Bim* [9] or the *1B-divalent metal transporter* (*1B-DMT1*) promoter [24].

The acetylation levels of both RelA and histones are regulated by histone deacetylases (HDACs) and histone acetyl transferases (HATs) [19]. The class I HDAC’s members (HDAC1-3) deacetylate NF-κB/RelA [25], while the atypical class III HDAC, sirtuin 1, selectively deacetylates the lysine 310 (K310) of RelA [8,26]. With the aim of restoring the altered acetylation of RelA and of histones after brain ischemia, we previously studied the combined association of entinostat (MS-275), a specific class I HDAC inhibitor, together with resveratrol, a sirtuin 1 activator [9]. MS-275 is a synthetic benzamide derivative under clinical evaluation for cancer therapy [27]. MS-275 has been demonstrated to inhibit HDACs1-3, and it is endowed with excellent pharmacokinetic properties [27]. The resveratrol mechanism of action includes the activation of the longevity factors SIRT1 [28] and AMP-activated kinase (AMPK), a serine-threonine kinase known to be a key metabolic and stress sensor/effector [29]. It has been shown that the small-chain-fatty-acid compound valproate inhibits the HDACs [30]. In particular valproate has major inhibitory activity on class I HDACs1,2,3 and 8 (millimolar range), while has no effects on HDACs 6,7 and 9 [31]. As a class I HDACs inhibitor, valproate could represent an alternative to MS-275, with the advantage to be suitable to the translational application in human brain ischemia, being routinely used to treat epileptic and bipolar patients. Notably, valproate was reported to be neuroprotective per se either in animal and cellular model of brain ischemia [32,33,34,35]. Clinical studies also demonstrated improved outcome in patients affected by intracerebral hemorrhage [36] or acute middle cerebral artery infarction when treated with the anti-epileptic doses of valproate [37]. On these premises, here we studied the effect of valproate in the association with resveratrol, both in the in vitro model of cortical neurons exposed to OGD and in mice subjected to transient “MCAO”.

## 2. Results

### 2.1. Valproate Elicits Synergistic Neuroprotection with Resveratrol in Mouse Cortical Neurons Exposed to OGD (Oxygen Glucose Deprivation)

**Figure 1:**
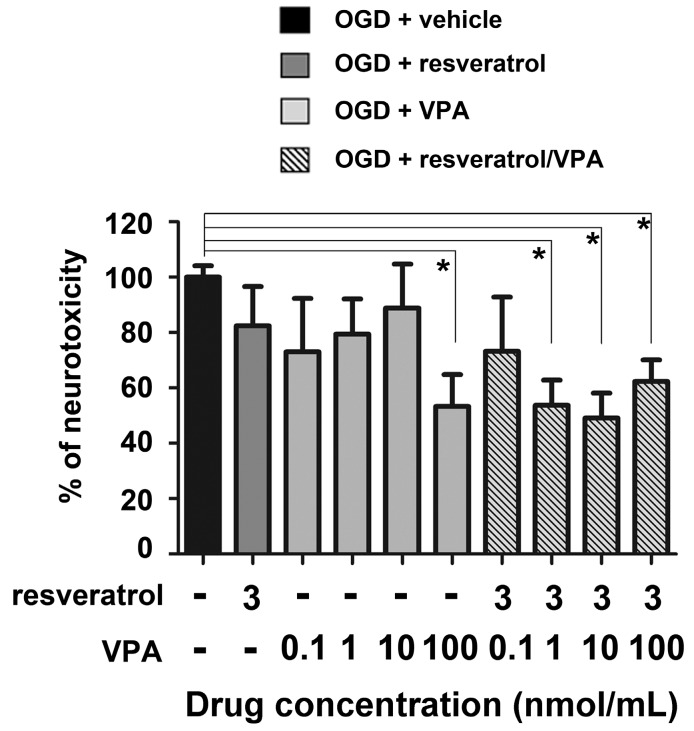
Valproate (VPA) and resveratrol elicited neuroprotective effects in primary cortical neurons exposed to oxygen glucose deprivation (OGD). VPA (100 nmol/mL), added during the 24 h recovery period, showed per se a significant neuroprotective activity. The combination of concentrations, per se ineffective, of resveratrol (3 nmol/mL) and VPA (1 nmol/mL) led to maximal neuroprotection. Values were expressed as a percentage of neurotoxicity, measured performing an lactate dehydrogenase (LDH) assay. Bars depicted the mean ± s.e.m. (Standard Error of the Mean) from three separate experiments run in triplicate. * *p* < 0.05 versus the corresponding OGD value. For statistical analysis one way ANOVA followed by Bonferroni post hoc test was performed.

We previously demonstrated that apoptosis preceded necrosis in OGD-exposed primary cultures of mouse cortical neurons. Within 2 and 6 h after OGD, neuronal cells displayed release of cytochrome c and terminal deoxynucleotidyl transferase dUTP nick end labeling (TUNEL)-positivity, respectively, in the absence of lactate dehydrogenase (LDH) release. The progressive release of cellular LDH became clearly detectable in the culture medium 24 h after the OGD, as an index of secondary necrosis [35]. In this experimental setting, the association of resveratrol and MS-275 elicited a synergistic neuroprotection [9]. The therapeutic potential of the drug association was confirmed in a MCAO model of brain ischemia [9]. By using both the same experimental approaches, we here tested the neuroprotective activity of resveratrol in association with valproate. Mouse primary cortical neurons, previously exposed to 3 h of OGD, were treated with valproate in the following 24 h. Valproate was tested at concentration ranging from 0.01 to 100 nmol/mL, alone or in association with 3 nmol/mL resveratrol. Valproate was neuroprotective per se at 100 nmol/mL concentration, while in association with resveratrol it was active at 1 nmol/mL concentration (Figure 1).

### 2.2. Association of Valproate and Resveratrol Normalizes the Acetylation State of Histones and RelA

**Figure 2:**
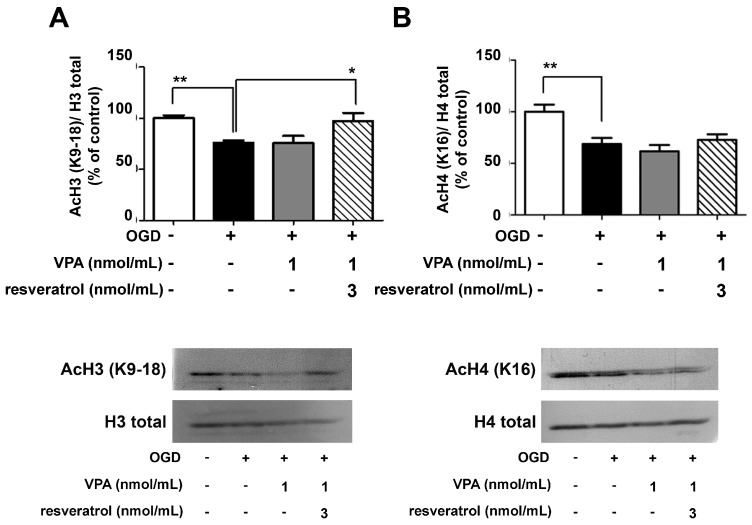
Western blots analyses of the H3 and H4 acetylation levels in nuclear extracts from OGD-exposed cortical neurons treated for 2 h with valproate (VPA) alone or in combination with resveratrol. (**A**,**B**) VPA (1 nmol/mL) did not increase per se the amount of H3 (K9/18) and H4 (K16) acetylation. Treatment with combined VPA (1 nmol/mL) and resveratrol (3 nmol/mL) completely restored the histone H3 acetylation. In the densitometry analysis of immunoblot bands data were expressed as percentage of the corresponding control value. Bars depicted the mean ± s.e.m. of three separate experiments, * *p* < 0.05 and ** *p* < 0.01 versus the corresponding OGD value.

We evaluated whether the neuroprotective effect of the drug combination was associated with the capability of valproate to inhibit neuronal HDACs, even at the low concentration used [30]. Western blot analyses of lysine acetylation at histones H3 and H4 were performed in nuclear extracts from cortical neurons exposed to OGD and treated with resveratrol (3 nmol/mL) and/or valproate (1 nmol/mL) in the following 2 h. In line with the effect observed by combining resveratrol and MS-275 [9], the combination of resveratrol and valproate restored the acetylation levels of H3 (K9/18), reduced by OGD (Figure 2A). The combined drugs did not increase the acetylation state of H4 (K16) (Figure 2B). As previously found with resveratrol alone (3 nmol/mL) [9], valproate alone (1 nmol/mL) did not affect histone acetylation.

**Figure 3:**
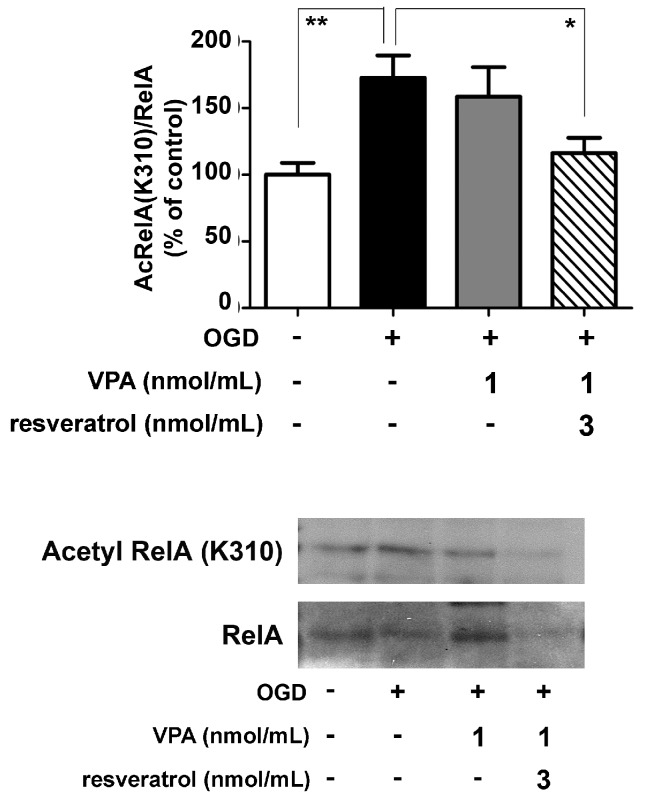
Effect of the valproate (VPA) and resveratrol combination on RelA acetylation state in OGD-exposed primary cortical neurons. Co-immunoprecipitation (co-IP) analysis of RelA acetylation in nuclear extracts of OGD-exposed cells treated during the following 2 h with vehicle or drugs. The combination of the drugs (VPA 1 nmol/mL and resveratrol 3 nmol/mL) site-specifically decreased the RelA K310 acetylation. In the densitometry analysis of immunoblot bands relative to RelA, data were expressed as percentage of the corresponding control value. Bars depicted the mean ± s.e.m. of three separate experiments, * *p* < 0.05 and ** *p* < 0.01 versus the corresponding OGD value.

Further co-immunoprecipitation (co-IP) analyses of nuclear extracts by specific anti-RelA antibodies were performed to investigate the level of RelA activation and acetylation. In line with previous evidence [8,9], OGD exposure induced the nuclear translocation of RelA as well as the RelA(K310) acetylation. Differently from resveratrol [9] or valproate alone, the combined application of the two drugs effectively reduced RelA(K310) acetylation (Figure 3).

### 2.3. Association of Valproate and Resveratrol Reduces the Activation of the Pro-Apoptotic Bim Promoter in Cortical Neurons

It has been reported that during OGD exposure of cortical neurons, the stimulation of Ac-RelA(K310) transactivates the pro-apoptotic *Bim* promoter [7,8,9]. Chromatin immunoprecipitation (ChIP) assays in cellular or animal models of brain ischemia also established that activated RelA binds the endogenous *Bim* promoter [9,23,24].

**Figure 4:**
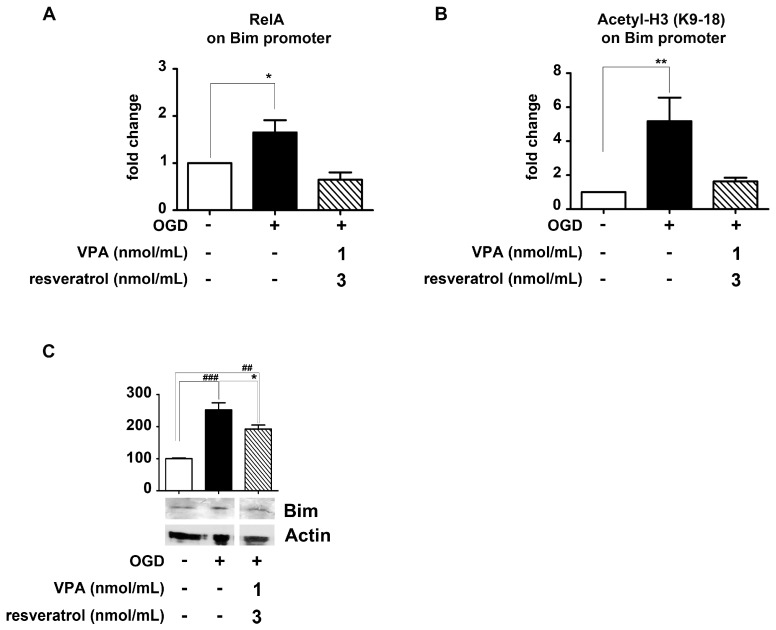
Effect of valproate (VPA) and resveratrol combination on RelA detachment from *Bim* promoter and reduction of Bim protein level, in primary cortical neurons exposed to OGD. (**A**,**B**) Cortical neurons were exposed to OGD and then treated 2 h with a combination of VPA (1 nmol/mL) and resveratrol (3 nmol/mL). Treatment with the drug combination significantly reduced the RelA binding to, and the H3 acetylation (K9/18) at, the *Bim* promoter. Results were obtained by real-time polymerase chain reaction (qRT-PCR) analyses of *Bim* promoter in immunoprecipitated DNA. Data were expressed as fold changes over values obtained in cells maintained in normal oxygen–glucose condition. Bars depicted the mean ± s.e.m. of three separate experiments, * *p* < 0.05 and ** *p* < 0.01 versus the corresponding control value; (**C**) Treatment with combined VPA (1 nmol/mL) and resveratrol (3 nmol/mL) significantly attenuated the Bim protein increase after OGD exposure. In the densitometry analysis of immunoblot bands data were expressed as a percentage of the corresponding control value. Bars depict the mean ± s.e.m. of three separate experiments, ## *p* < 0.01 and ### *p* < 0.001 versus the corresponding control value; * *p* < 0.05 versus the corresponding OGD value.

Likewise resveratrol and MS-275 association [9], the addition of resveratrol (3 nmol/mL) and valproate (1 nmol/mL) to the cells, in the 2 h after the OGD exposure, totally impaired the RelA binding at the *Bim* promoter as well as the consequent promoter-specific H3 (K9/18) acetylation (Figure 4A,B). These data correlated with the Bim protein level in the cytoplasm. After 2 h of recovery from the OGD, the Bim amount appeared increased, but it was reduced in cells exposed to resveratrol and valproate (Figure 4C).

### 2.4. Valproate Elicits Synergistic Neuroprotection with Resveratrol in Experimental Mouse Model of Brain Ischemia

**Figure 5:**
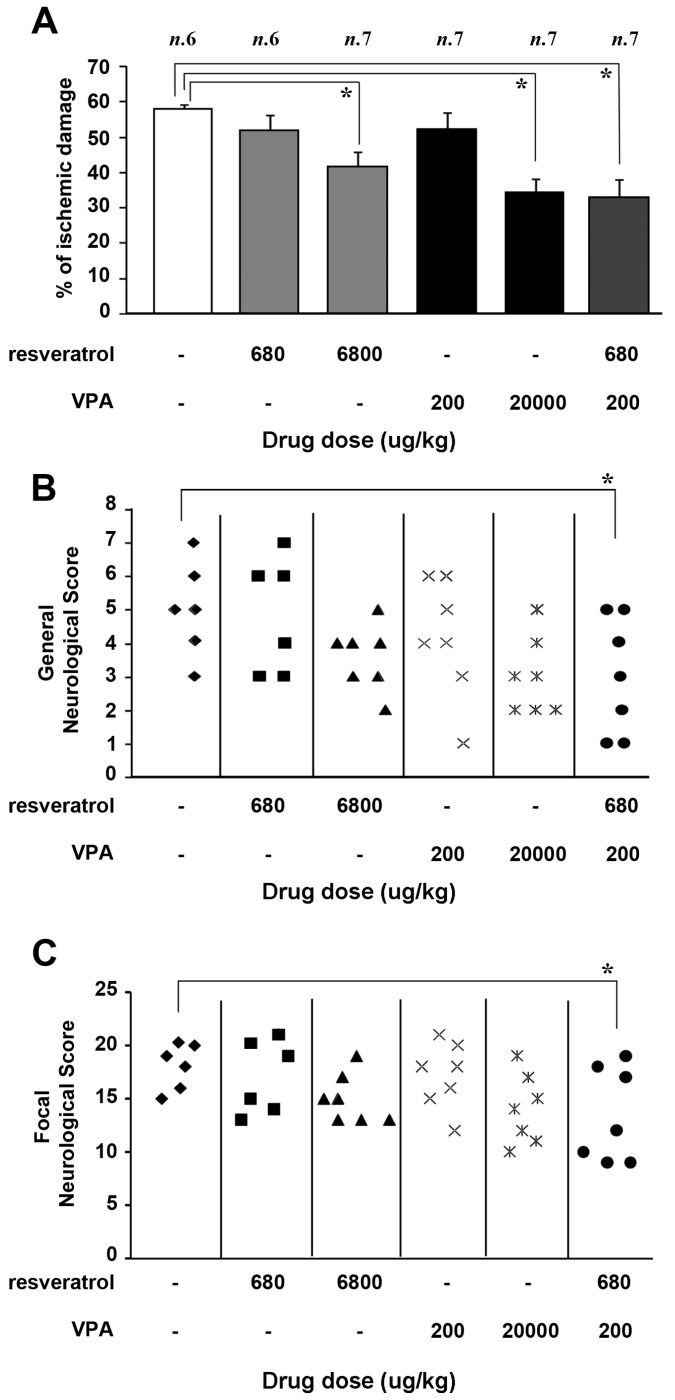
Effect of valproate (VPA), 200 and 20,000 µg/kg, and resveratrol, 680 and 6800 μg/kg, administered individually or in combination (VPA 200 μg/kg + resveratrol 680 µg/kg) in mice subjected to 60 min of middle cerebral artery occlusion (MCAO). Drugs were administered 30 min after the end of the MCAO period and evaluation of brain damage was performed 24 h after ischemia induction. (**A**) Effect of drug administration on ischemic damage. Each column represented the mean ± s.e.m. of the percentage of the infarct volume compared with the ipsilateral hemisphere. * *p* < 0.05 versus vehicle-treated group; (**B**,**C**) Effect of diverse doses of VPA and resveratrol, individually or in combination, on general and focal neurological scores, evaluated 24 h after the ischemia induction. * *p* < 0.05 versus vehicle-treated animals.

In mice subjected to 60 min of MCAO, the drugs were injected i.p. (intraperitoneally) 30 min after the beginning of the reperfusion period. Resveratrol was tested either at 680 or 6800 μg/kg. On the basis of 1:10 valproate-MS-275 relative potency [9] in in vitro neuroprotection (0.1 nmol/mL MS-275 vs. 1 nmol/mL valproate), valproate was tested in association with resveratrol at 200 µg/kg, a tenfold increase over the dose of MS-275 previously used in the synergistic combination with resveratrol [9] (Figure 5). Valproate was also tested alone at the antiepileptic dose of 20,000 μg/kg.

Twenty-four hours after ischemia, neurological function was scored in mice according to two scales: a general neurological scale and a focal neurological scale, as described in the Materials and Methods section [38,39]. The association of resveratrol 680 µg/kg and valproate 200 µg/kg significantly reduced the infarct volume (57.8 ± 1.8 vs. 32.8 ± 5, Figure 5A), as well as the neurological deficits (Figure 5B,C) when compared to vehicle treated animals. Worthy of note, on the basis of valproate pharmacokinetics [40], is the fact that the dose of 200 µg/kg i.p. in MCAO mice (Figure 5) was expected to produce a valproate brain concentration of ~1 nmol/mL, the same concentration that elicited synergistic neuroprotection with resveratrol in primary neurons exposed to OGD (Figure 1). When used separately at the same doses, neither the drugs produced neuroprotection (resveratrol 680 µg/kg: 51.6 ± 4.1; valproate 200 µg/kg: 52.2 ± 4.4, Figure 5A). Only when used at the higher doses (resveratrol 6,8 mg/kg or valproate 20 mg/kg) did they limit the infarct volume (resveratrol: 41.7 ± 3.9; valproate: 34.4 ± 3.8) and show a distribution of neurological scores slightly lower, although not statistically significant (Figure 5B,C).

## 3. Discussion

The present work gives preclinical evidence for the synergistic efficacy of resveratrol when administered with valproate, the old and largely used antiepileptic drug reported to inhibit the class I HDACs [30]. We here demonstrate that a valproate concentration of 1 nmol/mL elicited a synergistic neuroprotection with resveratrol (3 nmol/mL) in primary neurons exposed to OGD. When used alone, valproate was neuroprotective only at a concentration 100 folds higher (100 nmol/mL) than that effective in the synergy with resveratrol.

Different studies evidenced the increased histone acetylation as a pivotal epigenetic mechanism that promotes gene expression [41,42,43]. In line with previous evidence [9,44,45], we here confirmed the reduction of both histone H3 and H4 acetylation in cells exposed to OGD. This effect was reasonably related to a reduced HAT activity generated by the lower availability of acetyl-CoA, the fundamental co-factor for HAT enzymes, in response to the energy depletion due to the ischemic condition [46,47]. It has been demonstrated that the pharmacological inhibition of HDACs during brain ischemia increases histone acetylation [13]. This effect on the H3 histone was produced by either MS-275 [23] or valproate when used in association with resveratrol at very low concentrations. Neither valproate or MS-275 [9] or resveratrol [9] were able to increase the H3 acetylation when used individually at such low concentrations. Conversely, in neurons exposed to OGD, a single administration of resveratrol (3 nmol/mL) was found to be already effective in activating AMPK by promoting its phosphorylation at Thr 172 [9]. It is conceivable that AMPK, by inducing the catabolic pathways associated with ATP and NAD+ production contributes to the generation of acetyl-CoA in neuronal cells [48,49] which in turn can sustain the H3 acetylation in the presence of HDAC inhibitors, a mechanism that can contribute to the observed synergistic effect.

Valproate was not able to restore the histone H4 acetylation when combined with resveratrol at our experimental conditions. It could be explained by the short time (2 h) of recovery after OGD adopted in our investigation. Valproate showed to increase the histone H4 acetylation in rat models of brain ischemia, but only at later times from the onset of brain ischemia (72 h) and after repeated administration of the antiepileptic doses (300 µg/kg) [33,45]. An observation that would suggest a differential involvement of H3 and H4 acetylation in the early phases of neuroprotection.

NAD+, provided by AMPK, is the fundamental co-factor for sirtuins. Thus, AMPK may indirectly sustain sirtuin 1 activation by resveratrol [49]. As a proof of the involvement of AMPK-sirtuin pathway in the synergistic effect of resveratrol and HDAC inhibitors, either the compound C-mediated AMPK inhibition, or sirtuin 1 inhibition by sirtinol were found to abolish the neuroprotection induced by the combination of MS-275 and resveratrol in OGD-exposed neurons [9]. A recognized target of the deacetylase activity of sirtuin 1 [8,26] is RelA acetylated at the K310 residue and the association of resveratrol and valproate effectively reduced the RelA K310 acetylation. Clear-cut demonstration of the pro-apoptotic transcription mediated by acetylated RelA at the K310 has been previously provided by replacing lysine at the 310 residue with the acetylation-resistant arginine in mutagenesis experiments on RelA. Expressing the mutated RelA subunit in neurons abolished the OGD-mediated transcription of *Bim* [8] and *DMT1* [24] promoter luciferase plasmids and preserved the cell survival. ChIP analysis showed that either OGD in cells or lethal ischemia in mice induced the RelA binding and H3 (K9/18) histone acetylation at the endogenous promoters of *DMT-1* and *Bim* [8,9,24]. As previously reported for MS-275 and resveratrol [9], valproate and resveratrol totally hampered the binding of RelA, as well as the following H3 (K9/18) histone acetylation, at the *Bim* promoter in cultured neurons exposed to OGD. These effects correlated with a reduced cellular content of Bim protein. It can be inferred that as a result of 3 h of OGD exposure, the RelA binding at the *Bim* promoter increased the gene expression and the Bim protein levels. After the brief recovery (2 h) in the presence of the drugs, only the early phase of the protein drop to the control levels could be detectable, although its decrease was already significant.

We also demonstrated that administration of very low doses of valproate (200 µg/kg i.p.) and resveratrol (680 µg/kg i.p.) in mice subjected to MCAO significantly reduced infarct volume and neurological deficits. When used individually at the same low doses, neither the drugs produced neuroprotection. At higher doses they limited the infarct volume and induced a slight, not significant, reduction of neurological scores. Notably, in the synergistic combination with resveratrol, valproate reduced the neurological deficits at a dose at least a hundredfold lower than that commonly used in the seizure treatment [37], an aspect of the synergy that could unveil a much safer use of valproate for the clinical management of brain injury in post-ischemic patients. On the basis of pharmacokinetics evidence [40], the neuroprotective dose of valproate (200 µg/kg i.p) is expected to produce a valproate brain concentration of ~1 nmol/mL, the same concentration that we demonstrated to be neuroprotective with resveratrol in OGD-exposed primary neurons.

It can be concluded that valproate and resveratrol, by displaying a synergistic neuroprotective activity, may represent a promising, ready-to-use strategy for the therapy of post-ischemic brain damage.

Notably, a clinical study examining the use of valproate 400 mg/Bid versus placebo in post-stroke patients revealed no significant difference on the occurrence of comorbidities or seizure at one year. Though at one year it revealed a significant benefit in the secondary outcome of the National Institutes of Health Stroke Scale (NIHSS) score in the valproate-treated patients [36]. Also, a next open-label trial showed that post insult treatment with valproate (500 mg/Bid for three months) improved functional recovery in patients with acute middle cerebral artery infarction [37], suggesting a neuroprotective/neuro-remodeling effect of valproate when given at antiepileptic doses. Among valproate side effects, the most reported are dose-related and include tremor, gastrointestinal disturbances, bodyweight gain, liver toxicity, pancreatitis and neurological disorders [40]. By exploiting the combination with resveratrol, the present study discloses the translational potential to use valproate in post-stroke patients at much lower doses and drastically reduce the side effects of the drug in chronic treatments.

## 4. Material and Methods

### 4.1. Cell Culture

Primary cultures of mouse cortical neurons derived from 15-day-old embryonic mice, harvested with caesarean section from anaesthetized pregnant C57Bl/6 dams (Charles River, Italy) and cultured as previously described [50]. An initial plate density of 1.0 × 10^4^ cells/mm^2^ was used and 20 mm^2^ culture dishes were used for the viability studies, 210 mm^2^ culture dishes for Western blot and co-immunoprecipitation analyses and 560 mm^2^ culture dishes (Nunc, Langenselbold, Germany) for chromatin immunoprecipitation (ChIP) assays. All experiments were carried out at 11 days in vitro (DIV).

### 4.2. Oxygen Glucose Deprivation and Measurement of Lactate Dehydrogenase (LDH) Release

Primary cortical neurons at the eleventh DIV were exposed to OGD for 3 h, as previously described [50]. Control cells were incubated in a normal aerated incubator for the same time period. Cells recovered for 24 h in culture medium (Neurobasal medium containing 0.4% B27 supplement) typically aerated in the incubator. At the end of the recovery, neuronal injury was evaluated by measuring the amount of lactate dehydrogenase (LDH) (Promega, Madison, WI, USA) released relative to total releasable LDH. Different concentrations of valproate (from 0.1 to 100 nmol/mL) or resveratrol (3 nmol/mL) alone or combined were added to the cells after the OGD period. Valproate (Sigma-Aldrich, Saint Louis, MO, USA) was dissolved in recovery medium and resveratrol (Merck KGaA, Darmstadt, Germany) in dimethyl sulfoxide (DMSO) and diluted to a final DMSO concentration lower than 0.3%. Control cells were treated with vehicle.

### 4.3. Co-Immunoprecipitation and Western Blot Analyses

Immunoprecipitation and Western blot studies of RelA and histone acetylation were performed in nuclear protein extracts prepared as previously described [50] from primary cortical neurons exposed to 3 h of OGD and maintained 2 h in recovery medium in presence or absence of valproate (1 nmol/mL) and/or resveratrol (3 nmol/mL). Briefly, a total of 40 μg of nuclear extracts were incubated at 4 °C overnight with 2 μg/μL of goat polyclonal anti-RelA antibody (sc-372GX, Santa Cruz Biotechnology, Santa Cruz, CA, USA) and co-immunoprecipitated proteins were detected by immunoblotting. The following antibodies were used: rabbit polyclonal anti-RelA (1:100, sc-372, Santa Cruz Biotechnology), rabbit polyclonal anti-Acetyl-RelA (Lys310) (1:500, #3045, Cell Signaling, Danvers, MA, USA).

For Western blot analyses, nuclear proteins (15–20 μg proteins/sample) were resolved by Bolt™ 4–12% Bis-Tris Plus SDS/polyacrylamide pre-cast gels (Invitrogen, Waltham, MA, USA). Immunodetection was performed by incubating the membrane overnight at 4 °C, with the following primary antibodies: rabbit anti-H3 (#9715 Cell Signaling), rabbit anti-Acetyl H3 (K9-18) (#07-593 Upstate-Millipore, Billerica, MA, USA), rabbit anti-H4 (#07-108 Upstate-Millipore), rabbit anti-Acetyl H4 (K16) (#06-762 Upstate-Millipore).

For western blot analyses of Bim protein 30 µg of cytosolic extracts were resolved by Bolt™ 10% Bis-Tris Plus SDS/polyacrylamide pre-cast gels (Invitrogen). Immunodetection was performed by incubating the membrane overnight at 4 °C, with the following primary antibodies: rabbit anti-Bim (#14-6265 eBioscience, Waltham, MA, USA) and mouse anti-actin (#A4700 Sigma-Aldrich).

Quantification of protein expression was performed by densitometry analysis of immunoblots, using Gel Pro.3 analysis software (MediaCybernetics, Rockville, MD, USA).

### 4.4. Chromatin Immunoprecipitation Assay and Real-Time PCR Analysis

Chromatin immunoprecipitation (ChIP) assays were performed in primary cortical neurons as previously described [9]. The ChIP assay kit (#9003S, Cell Signaling Technology) was used to study H3 histone acetylation and RelA binding at the *Bim* promoter. Primary cultures of mouse cortical neurons were exposed to 3 h of OGD and then treated with valproate (1 nmol/mL) and resveratrol (3 nmol/mL) for 2 h in recovery medium. The sheared chromatin was incubated with anti-acetyl H3 (K9/18) or anti-RelA or anti-IgG (negative control) overnight at 4 °C. An aliquot of chromatin, not incubated with antibody, was used as the input control sample. Antibody-bound protein/DNA complexes was washed, eluted, treated with proteinase digest proteins, and subjected to real-time polymerase chain reaction (qRT-PCR) analyses.

The specific primers used to amplify the mouse *Bim* promoter were as follows:

forward, 5-CTGGATGCAGGTTGGGTAG-3
reverse, 5-GGGAATGAGAAAGTTAGCTGGA-3

These specific primers generated a 410-bp product. Incorporation of the SYBR Green dye into the PCR products was monitored in real-time with a ViiA™ 7 Real-Time PCR detection system (Applied Biosystems, Waltham, MA, USA), allowing the determination of the threshold cycle (*C*_t_) at which the exponential amplification of PCR products began.

### 4.5. Transient Middle-Cerebral Artery Occlusion Model (MCAO)

Mice were subjected to MCAO as previously described [51,52]. A 5-0 nylon filament was inserted through the external carotid artery stump and advanced into the left internal carotid artery until it blocked the origin of the middle cerebral artery (MCA). After 60 min MCA occlusion, the filament was withdrawn to restore blood flow. Body temperature was monitored throughout the entire duration of the surgical procedure and maintained at 37.5 °C with a thermostatic blanket. Experiments were performed according to the international guidelines for animal research and approved by the Animal Care Committee of “Federico II” University of Naples, Italy and by Italian Minister of Health (11 May 2015), Autorization n. 355/2015-PR.

### 4.6. Monitoring of Blood Gas Concentration and Cerebral Blood Flow (CBF) with Laser-Doppler Flowmetry

A catheter was inserted into the femoral artery to measure arterial blood gases before and after ischemia (Rapid lab 860; Chiron Diagnostic, Medfield, MA, USA). CBF was monitored in the cerebral cortex ipsilateral to the occluded MCA with a laser-doppler flowmeter (Periflux system, 5000, Perimed AB, Järfälla, Sweden) [53]. Once a stable CBF signal was obtained, the MCA was occluded. CBF monitoring was continued up to 30 min after the end of the surgical procedure once the occurred reperfusion was verified.

### 4.7. Evaluation of Ischemic Volume and Neurologic Deficit Scores

Mice were decapitated 24 h after ischemia. Ischemic volume was evaluated by 2,3,5-triphenyltetrazolium chloride staining [54]. The brains were cut into 500 μm coronal slices with a vibratome (Campden Instrument, 752 M, Loughborough, UK). Sections were incubated in 2% 2,3,5-triphenyltetrazolium chloride for 20 min and in 10% formalin overnight. The infarcted area was calculated by image analysis software (Image-Pro Plus, Rockville, MD, USA) [55]. The total infarct volume was expressed as a percentage of the volume of the hemisphere ipsilateral to the lesion. In mice, 24 h after ischemia, neurological function was scored according to two scales: a general neurological scale and a focal neurological scale, as described by Clark et al. (1997) [38]. In the general score, six general deficits were measured: (a) hair conditions (0–2); (b) position of ears (0–2); (c) eye conditions (0–4); (d) posture (0–4); (e) spontaneous activity (0–4), and; (f) epileptic behavior (0–12). For each of the six general parameters measured, animals received a score that correlated directly with the degree of symptom severity, as previously reported [38,39]. The scores of investigated items were then summed to provide a total general score ranging from 0 to 28. For the focal score, seven areas were assessed: (a) body symmetry; (b) gait; (c) climbing; (d) circling behavior; (e) front limb symmetry; (f) compulsory circling; and (g) whisker response. The severity of each of these items was rated on a scale from 0 to 4. The seven items were then summed to give a total focal score ranging between 0 and 28. The single item does not provide any significant information per se. A higher score correlated with the worst animal condition [38,39]. Ischemic volume, neurologic function, and animal survival were evaluated in a blinded manner.

### 4.8. Experimental Protocol

Valproate and resveratrol were dissolved in saline and in dimethyl sulfoxide (DMSO) respectively and administered intraperitoneally (i.p.) 30 min after the beginning of the reperfusion. The doses used were 680 and 6800 μg/kg for resveratrol and 200 μg/kg and 20 mg/kg for valproate. Control mice undergoing MCAO received vehicle solution in the same volume and on the same time schedule as the valproate or resveratrol-treated animals. The doses used in vivo were chosen in the attempt to obtain in the cerebrospinal fluid (CSF, 50 μL) pick concentrations that was effective on infarct in our previous paper [9,56]. By considering that only 2% of plasmatic resveratrol can cross the blood brain barrier (BBB) [56], to obtain a resveratrol concentration of 3 nmol/mL in the CSF, we injected a dose of 68 μg/kg as starting dose. In present paper, we tested a dosage of 680 μg/kg in order to replicate the dosage scaling that led us to identify the resveratrol therapeutic dose of 6800 μg/kg. We also approximated a total absorption through the i.p. route of administration and a compensation of drug metabolism by increased BBB permeability after brain ischemia. Dosages of valproate were chosen in order to predict an initial CSF concentration thirty times lower than that of resveratrol. We also administered the drugs at 100-fold higher doses. Mice were sacrificed 24 h after the MCAO. Body temperature, PaO_2_, PaCO_2_ and pH values were monitored for the entire experiment both in control and drug-treated groups. Animals were randomly assigned either to the vehicle or the compound treatment groups.

### 4.9. Statistical Analysis

Data obtained in cultured neurons were expressed as mean ± standard error and statistical significance of differences between groups was evaluated by one-way ANOVA followed by Dunnet’s multiple comparison test, using GraphPad Prism 5 software (GraphPad Software, Inc., La Jolla, CA, USA, version Prism 5). *p* < 0.05 was considered to be significant.

## Abbreviations

tPA	Tissue plasminogen activator
HAT	Histone acetyltransferase
HDAC	Histone deacetylase
MCAO	Middle cerebral artery occlusion
OGD	Oxygen glucose deprivation
AMPK	AMP-activated kinase
ChIP	Chromatin immunoprecipitation
DIV	Days in vitro
LDH	Lactate dehydrogenase
DMSO	Dimethyl sulfoxide
qRT-PCR	Real-time polymerase chain reaction
CBF	Cerebral blood flow
CSF	Cerebrospinal fluid
BBB	Blood brain barrier
